# Model-based extended quaternion Kalman filter to inertial orientation tracking of arbitrary kinematic chains

**DOI:** 10.1186/s40064-016-3653-8

**Published:** 2016-11-14

**Authors:** Agnieszka Szczęsna, Przemysław Pruszowski

**Affiliations:** Institute of Informatics, Silesian University of Technology, Akademicka 16, 44-100 Gliwice, Poland

**Keywords:** Inertial motion capture, Orientation estimation, Kalman filter, Quaternion, Kinematic chain

## Abstract

Inertial orientation tracking is still an area of active research, especially in the context of out-door, real-time, human motion capture. Existing systems either propose loosely coupled tracking approaches where each segment is considered independently, taking the resulting drawbacks into account, or tightly coupled solutions that are limited to a fixed chain with few segments. Such solutions have no flexibility to change the skeleton structure, are dedicated to a specific set of joints, and have high computational complexity. This paper describes the proposal of a new model-based extended quaternion Kalman filter that allows for estimation of orientation based on outputs from the inertial measurements unit sensors. The filter considers interdependencies resulting from the construction of the kinematic chain so that the orientation estimation is more accurate. The proposed solution is a universal filter that does not predetermine the degree of freedom at the connections between segments of the model. To validation the motion of 3-segments single link pendulum captured by optical motion capture system is used. The next step in the research will be to use this method for inertial motion capture with a human skeleton model.

## Background

Inertial motion capture systems are based on body sensor network, where inertial measurement unit (IMU) sensors are attached to each major segment that should be tracked (Kulbacki et al. 2015; Roetenberg et al. 2009). The model (skeleton) of tracked object is built from rigid-body segments (defined as bones) connected by joints. The mapping of IMU orientations to specific segments of the body model allows for motion capture of the subject. The orientations of sensors are typically estimated by fusing a gyroscope rate ($\omega$), linear acceleration (*a*), and magnetic field measurements (*m*) with respect to the global reference frame (usually aligned with Earth gravity and local magnetic north). With knowledge of all the orientations of segments over time, the overall pose can be tracked. In the literature, many methods for orientation estimation based on single IMU output signals can be found e.g., Kalman filters (Sabatini 2011; Madgwick et al. 2011), complementary filters (Mahony et al. 2008). However, this loosely coupled approach where each segment is treated independently has numerous drawbacks. Joint constraints, such as those found in the human anatomy, cannot be included easily into the tracking. The correlations between the segments are lost during estimation (Miezal et al. 2013). Furthermore, tightly coupled systems, where all parameters and measurements are considered jointly in one estimation problem, have previously been shown to provide better performance (Young 2010).

In Young et al. (2010), propagation of linear accelerations through the segment hierarchy was used to improve the identification of the gravity components under high acceleration motions. That solution is based on a very simple complementary filter (Szczęsna et al. 2016).

Next, we can find solutions based on the Kalman filter for a specific set of segments by predetermining the DOF at the connections between the segments of the model. Such solutions are based on the Denavit–Hartenberg convention and use Euler angles as their orientation representation. Examples are the extended Kalman filter for lower body parts (hip, knee, ankle) (Lin and Kulić 2012) and the unscented Kalman filter with similar process and measurement model for shoulder and elbow joint angle tracking (El-Gohary and McNames 2012).

In Šlajpah et al. (2014), authors propose extended Kalman filters for each segment using 18 element state vectors. This algorithm uses quaternion representation of orientation. The solution is limited only to human walking.

A different concept is presented in Vikas and Crane (2016) where the joint angle is estimated based on more than one sensor, placed on the segment. The system is based on vestibular dynamic inclination measurements and estimates only 2 Euler angles.

Multibody systems based on IMU sensors can also estimate and track other parameters like positions, velocities, and accelerations (Torres-Moreno et al. 2016).

The reported errors in the aforementioned publications (Young et al. 2010; Lin and Kulić 2012; El-Gohary and McNames 2012; Šlajpah et al. 2014) are not comparative because experiments had different conditions and concerns about various movements and errors were calculated in an inconsistent way, all of the average angles errors were about 4°–7°. The referenced values were obtained from different sources: simulated, mechanical, optical systems or calculated based on depth camera.

This paper proposes a new model-based extended quaternion Kalman filter (MBEQKF) that allows estimation of orientation on the basis of outputs from the IMU sensors. This filter reflects interdependencies from the construction of the kinematic chain so that the orientation estimation is more accurate. The proposed solution is a universal filter that does not predetermine any degree of freedom (DOF) at the connections between the segments of the model. Our aspiration for future work is to use our novel method for inertial motion capture.

## Model-based extended quaternion Kalman filter (MBEQKF)

The aim was to simplify the structure of the filter while maintaining corrections resulting from the kinematic relationships in the model; another important element was versatility. The proposed solution does not predetermine any DOF at the connections between the segments of the model, as it is in solutions based on the Denavit–Hartenberg convention (El-Gohary and McNames 2012; Lin and Kulić 2012).

As a base of implementation, the quaternion extended Kalman filter with direct state was used. The unit quaternion $q = (q_0, [q_1, q_2, q_3])^T\,\epsilon\,\mathbb {H}$ represents the body orientation, where $\mathbb {H}$ is a four-dimensional non-commutative division algebra over the real numbers. The orientation quaternion is MBEQKF filter state vector $x = q$. The angular velocity is considered to be a control input (like in Angelo 2006). The angular velocity is not part of the state vector so model of dynamic, e.g., dynamic model of human limb motion in terms of first-order Gauss–Markov stochastic process (Yun and Bachmann 2006), is not in the development of the filter equations. The state vector has a smaller dimension and it is not necessary to include first and second derivative of angular velocity in the state vector to obtain the optimal model (Sabatini 2011; Sabatini 2011; Foxlin 1996), like in filter described in Šlajpah et al. (2014) where the state vector has 18 and measurement vector has 12 elements. The non-linear measurement equations are defined by rotating the reference vectors (magnetic Earth field and gravity) using estimated orientation quaternion. Also, the Newton–Euler kinematic motion equations are used to model acceleration measurements in the kinematic chain.

The MBEQKF filter process model is kinematic Eq. (1) (Chou 1992), which describes the relation between temporal derivatives of an orientation represented by unit quaternion *q* and angular velocity of the body frame ($^{B}{\omega }$) relative to the global frame *N*:

1 $$\begin{aligned} \frac{d}{dt}q(t)=\frac{1}{2}q(t)\otimes (0, ^{B}{\omega })^T \end{aligned}$$

where $\otimes$ stands for the quaternion multiplication. The left superscript indicates that the coordinate frames in which vectors are expressed (measured) are *N* for the Earth fixed coordinate system or *B* for the system related to the moving body.

Multiplying two unit quaternions gives a unit quaternion representing the composition of the two rotations. Hence, we can describe the orientation now and at the next moment in time, assuming a constant angular velocity:

2 $$\begin{aligned} q(0) = q_0,\quad q(1) = q_k \otimes q_o,\quad q(t) = (q_k)^t \otimes q_o \end{aligned}$$

By using Euler formula for quaternion we can write quaternion as $q_k = exp(\frac{\theta }{2}n)$, where $\theta$ is an angle, and *n* is the unit-vector axis of rotation. Where $\theta n$ represent instantaneous angular velocity.

The resulting quaternion produced by the process must be normalized. We used the brute-force approach and normalise the quaternion after the measurement update stage, which lead to a suboptimal algorithm (Sabatini 2011).

By using the orientation quaternion, every vector can be translated from the global frame *N* to the body coordinate system *B*:

3 $$\begin{aligned} ^{B}{v}= q^{*}\otimes (0, ^{N}{v})^T\otimes q \end{aligned}$$

and from body to global

4 $$\begin{aligned} ^{N}{v}= q\otimes (0, ^{B}{v})^T\otimes q^{*} \end{aligned}$$

where $q^*$ is a conjugate quaternion:

$$\begin{aligned} q^*=(q_0, [-q_1, -q_2, -q_3])^T \end{aligned}$$

In practical implementations, orientation estimation is realised on the basis of digital systems. The discrete time index is denoted by the subscript *k*. The discretized priori state estimation equation of the orientation kinematics process corresponding to (Eqs.1 and 2) is as follows:

5 $$\begin{aligned} x^{-}_{k+1}=\varPhi _{k}x_{k}+n_{k}=\exp \left[ \frac{1}{2}M_{R}(^B{\omega }_k)\varDelta t\right] x_{k}+n_{k}. \end{aligned}$$

In this equation, $x^{-}_{k}$ is the discrete—priori estimates time state vector, $x^{-}_{k}=q_{k}$, and $M_{R}(^B{\omega }_k)$ denotes matrix representation of the quaternion right multiplication corresponding to the pure quaternion $(0, ^B{\omega }_k)^T$, and $\varPhi$ is the state transition matrix. The components of the state vector are modelled as random walk, where *n* is the zero-mean white noise process with covariance matrix $\sigma _{g}^{2}I$. The quaternion time-evolution is a first-order approximation of the exact process (1).

As the gyroscope data are external inputs to the filter rather than measurement, gyroscope measurement noise enters the filter as process noise through a quaternion-dependent linear transformation (Sabatini 2011). The process noise covariance matrix $Q_{k}$ is following:

6 $$\begin{aligned} Q_{k}=(\varDelta t/2)^{2}\varXi _{k}(\sigma _{g}^{2}I_{4x4})\varXi _{k}^{T} \end{aligned}$$

where for $q_k=(a, [b,c,d])$ we define as follows

$$\begin{aligned} \varXi _k=\left[ \begin{array}{ccc} a&\quad-d&\quad c\\ d&\quad a&\quad -b\\ -c&\quad b&\quad a\\ -b&\quad -c&\quad -d\\ \end{array} \right] \end{aligned}$$

The model of tracked object is built from rigid-body segments connected by joints. Sensors are attached to segments with constant offset vector from the centre of segment rotation. The defined model is a skeleton of object. In our experiments we use model of 3-segments pendulum. Each segment (with IMU) and joint has a local coordinate frame related to the coordinate frame of the sensor. Joints form a hierarchy structure with the position of a child joint given by an offset from the parent joint centre. Resulting orientations are calculated in the world coordinate frame based on two reference vectors, Earth gravity *g* and magnetic north *mg*. Quantities marked with superscript *j* are referring to a corresponding *j* segment.

The Newton–Euler equations describe the combined translational and rotational dynamics of a rigid body and can be the base of the measurement model of acceleration in a kinematic chain (skeleton model). The modelled linear acceleration of the sensor is considered the case of a rigid body rotating about a point, fixed at the origin with angular velocity $\omega$. Every point on this body will have a radial linear acceleration:

7 $$\begin{aligned} l_r = (\omega \cdot o)\omega - o \left\| \omega \right\| ^2 \end{aligned}$$

where *o* is the offset of the point from the centre of rotation.

Also the every point on the rigid body has tangential acceleration:

8 $$\begin{aligned} l_t = \alpha \times o \end{aligned}$$

where $\alpha$ is an angular acceleration calculated from angular velocity as:

9 $$\begin{aligned} \alpha = \frac{\omega _{k+1} - \omega _{k-1}}{2\varDelta t} \end{aligned}$$

The angular acceleration is the derivative of angular velocity and can be calculated for example by first central difference approximation based on angular velocity samples.

The whole body is in a rotating frame with a linear acceleration $l_f$ and this is a linear acceleration from the parent segment in the skeleton model. The resulting linear acceleration of a point under that assumption is therefore: $l = l_f + l_r + l_t$.

In the model for every segment we have a linear acceleration of the sensor $a^{S,j}$ (10), and a linear acceleration of the joint $a^{J,j}$ (11), which is passed to the next segment $j+1$ as a linear acceleration of the parent. All linear accelerations that are passed between segments are in the global coordinate frame $^{N}{a}^{J,j}_k$.

The model sensor acceleration is:

10 $$\begin{aligned} ^{B}{a}^{S,j}_{k}\,=\,^{B}{a}^{J, j-1}_{k} +\, ^B{\omega }_{k}\,\times\,(^B{\omega }_{k} \times ^B{o}^{S,j}_{k}) +\,^{B}{\alpha }_k\,\times\, ^B{o}^{S,j}_k +\, ^B{g}_{k} \end{aligned}$$

The model joint acceleration is:

11 $$\begin{aligned} ^B{a}_k^{J,j}\,=\, ^{B}{a}^{J, j-1} +\, ^B{\omega }_{k} \times (^B{\omega }_{k} \times ^B{o}^{J,j}_k) +\, ^{B}{\alpha }_k \,\times\, ^B{o}^{J,j}_{k} \end{aligned}$$

where

12 $$\begin{aligned} ^{B}{a}^{J, j-1}_k &= q_k^{*}\otimes (0, ^{N}{a}^{J,j-1}_k)^T\otimes q_k \end{aligned}$$

13 $$\begin{aligned} ^B{g}_k& = q_k^{*}\otimes (0, ^{N}{g})^T\otimes q_k \end{aligned}$$

14 $$\begin{aligned} ^{N}{a}^{J, j}_k& = q_k\otimes (0, ^{B}{a}^{J,j}_k)^T\otimes q_k^{*} \end{aligned}$$

Also, the distances must be transformed into the body coordinate frame:

15 $$\begin{aligned} ^{B}{o}_k = q_k^{*}\otimes (0, ^{N}{o}_k)^T\otimes q_k \end{aligned}$$

The MBEQKF filtering algorithm use model (5) for predicting aspects of behaviour of a system and a model of the sensor measurements (16), in order to produce the most accurate estimation of the state of system. The resulting measurement model, based on a priori estimates of the state vector, is of the form:

16 $$\begin{aligned} f(x^{-}_{k} = q_k)= \begin{bmatrix}^B{a}^{S,j}_k\\ q_k^{*}\otimes (0, ^{N}{mg})^T\otimes q_k \end{bmatrix}+\begin{bmatrix}n_{k}^{a}\\ n_{k}^{m} \end{bmatrix} \end{aligned}$$

where $n_{k}^{a}$ and $n_{k}^{m}$ are the accelerometer and magnetometer measurement noise with covariance matrices $\sigma _{a}^{2}I$ and $\sigma _{m}^{2}I$. The measurement noise covariance matrix *V* represents the level of confidence placed in the accuracy of the measurements:

17 $$\begin{aligned} V = \begin{bmatrix}\sigma _{a}^{2}I&\quad 0\\ 0&\quad\sigma _{m}^{2}I \end{bmatrix} \end{aligned}$$

Since the above output is non-linear, it is linearized by computing the Jacobian matrix,

18 $$\begin{aligned} H_{k}=\frac{d}{dx_{k}}f({x_k})\left| _{x_{k}=x^{-}_{k}}\right. . \end{aligned}$$

According to the notation introduced above, the our filter MBEQKF equations are summarised as follows:

- the priori state estimate is:
  19 $$\begin{aligned} x^{-}_{k+1}=\varPhi _{k}x_{k} \end{aligned}$$
- the priori error covariance matrix is:
  20 $$\begin{aligned} P^{-}_{k+1}=\varPhi _{k}P_{k}\varPhi _{k}^{T}+Q_{k} \end{aligned}$$
- the Kalman gain is:
  21 $$\begin{aligned} K_{k+1}=P^{-}_{k+1}H_{k+1}^{T}(H_{k+1}P^{-}_{k+1}H_{k+1}^{T}+V_{k+1})^{-1} \end{aligned}$$
- the posteriori state estimate is:
  22 $$\begin{aligned} x_{k+1}=x^{-}_{k+1}+K_{k+1}[z_{k+1}-f(x^{-}_{k+1})] \end{aligned}$$
  The proposed filter is an additive filter which relaxes the quaternion normalization condition and treats the four components of the quaternion as independent parameters and uses the addition operation. Next, the resulting quaternion is normalized.
- the posteriori error covariance matrix is:
  23 $$\begin{aligned} P_{k+1}=P^{-}_{k+1}-K_{k+1}H_{k+1}P^{-}_{k+1}. \end{aligned}$$

## Experimental set-up

For a test of the proposed universal MBEQKF, a 3-segment single linked pendulum was built. As reference, data from the optical system of motion capture (Vicon system) were used. Experiments demonstrate that using body model kinematic dependences in the orientation filter can improve the accuracy of the inertial motion capture system. Through simple procedural calibration and mounting sensors permanently, we remove the effect of bad calibration factors on the estimation of orientation.

**Fig. 1:**
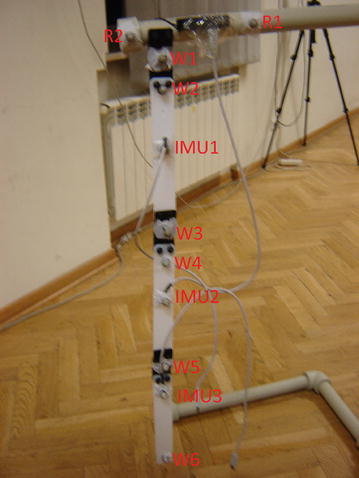
The 3-segment pendulum with 3 IMU sensors marked as IMU1, IMU2 and IMU3 and markers for optical system marked as R1, R2, W1, W2, W3, W4, W5, and W6

**Fig. 2:**
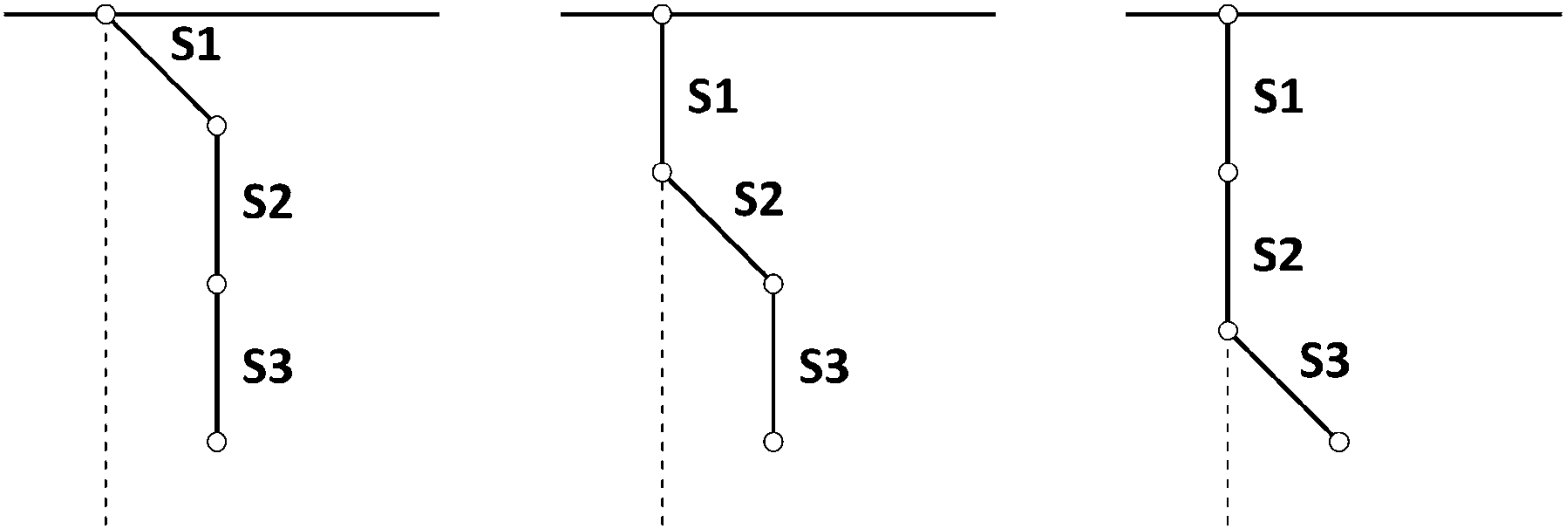
The model of pendulum made up of 3 segments (S1, S2, S3) connected by single links with extorsion angles to segments S1, S2 and S3 used during tests

The pendulum was built with three segments connected by movable single linked joints. An IMU sensor built at the *Silesian University of Technology, Department of Automatic Control and Robotics* was fixed to each segment. The published in Jedrasiak et al. (2013) IMU sensors signal to noise coefficients are as follows: accelerometer 43.2, magnetometer 767.9 and gyroscope 254.5. These IMU sensors have been marked as IMU1, IMU2 and IMU3 (Figs. 1, 2). On the pendulum, markers were also attached, marked as R1, R2, W1, W2, W3, W4, W5, and W6. The motion of the pendulum was mainly on one axis, but motion on other axes was also measured (shaking and swinging the pendulum from side to side). This had no effect on the results of estimations because the axes of motion are not aligned with the sensor axes.

The data were recorded by using a USB connection of sensors to the PC via application, which allowed for the capture of raw signals from IMU sensors. Next the data were processed by filters implemented in Matlab.

Recordings were captured with seven different scenarios (each scenario repeated 3 times) carried out using the Vicon system with a frequency of 100 Hz. The IMU sensors also worked with such a frequency. The recordings had a length from 9600 to 16228 samples. The recorded movement is characterized by different values of acceleration amplitudes: (4–15> $\rm{m /s^2}$ for low acceleration dataset and (15–23> $\rm{m /s^2}$ for high acceleration dataset. The optical system also enabled calibration of sensors and calculation of the necessary distances. The scenarios relied on forcing motion (Low or High swing) to a certain segment (S1—up, S2—middle, and S3—down) of the pendulum (Table 1). The initial extorsion angles to each segment are presented in Fig. 2. The scenario marked as Dynamic relied on repeated forcing swing and recording to the total suppression of the pendulum. The data are available in our RepoIMU repository^1^ (Szczęsna et al. 2016).

**Table 1:** Description of experiments data

Name	Samples number	Initial extorsion (°)	Max. $\omega$ (rad /s)	Max. amplitude *a* ($\rm{m/s^2}$)
*High accelerations*				
Middle_Hight	14805	90 (to S2)	9	15.4
Up_Hight	9769	90 (to S1)	10.5	22
Dynamic	16228	9 times repeated 90 (to S1)	11	23
*Low accelerations*				
Down_Low	10051	45 (to S3)	2.7	4
Middle_Low	9621	45 (to S2)	4.6	4.2
Up_Low	9921	45 (to S1)	10.3	13.4
Down_Hight	12799	90 (to S3)	6.6	11

### Data synchronization and error calculation

Each experiment was recorded using Vicon Nexsus system with a sampling frequency of 100 Hz. In order to provide an informative comparison of orientation data streams with different reference frames and measured according to separate timers with the same frequency, the data must be normalized. Such a procedure can be divided into two steps: normalization in the time domain (time synchronization) and transformation of orientations to the same reference frame.

Transforming one orientation data stream from one reference frame to the other one is a simple geometric operation—rotation. Only knowledge about the relationship between two world reference frames—navigation and body—is required. As a reference body frame, the first body frame from the time domain was chosen.

Signals from the Vicon system and IMU sensors were captured at the same frequency. Knowing that, in order to synchronize the time domain we needed to find the time offset $(\varDelta {t})$ between the two signals. A time window was chosen to be $<-\varDelta t^{Max} , \varDelta t^{Max}>$, where $\varDelta t^{Max}$ is the maximal offset we expected $(-\varDelta t^{Max}< \varDelta t < \varDelta t^{Max})$. The distance between the two signals for each time offset in the window is calculated. Synchronization is performed on the $^B\omega _{IMU}$ signal. The Vicon system does not calculate angular velocity of the body directly, so it must be calculated by the equation:

24 $$\begin{aligned} \omega _{Vicon}= 2 * {q}_{Vicon}^{-1} \otimes \frac{dq_{Vicon}}{dt} \end{aligned}$$

where $q^{-1}$ is the inverse of *q*.

Evaluation of performances of the presented filter was done on the basis of the average deviations between true and estimated orientations of the body (Gramkow 2001). Here, we used the deviation index *DI* corresponding to the geodesic distance between two quaternions—filter estimate $\hat{q}$ and the true rotation *q* from the Vicon system, on the hypersphere $S^3$:

25 $$\begin{aligned} DI=2 * arccos(| \hat{q} * q |) \end{aligned}$$

All evaluations and comparisons of the performances of algorithms for orientation estimation are based on the deviation index averaged over the experiment time horizon.

### Filter parameters

Filter parameters are following:

- reference vectors: $^{N}g=[0, 0, -9.81]^{T}$ and $^{N}m=[\cos (\varphi ^{L})-\sin (\varphi ^{L})]^{T},$ where $\varphi ^{L}$ is the geographical latitude angle. For the geographical position of the laboratory, where measurements were done, we have $\varphi ^{L}=66^{\circ}=1.1519$ rad;
- parameters of noise: $\sigma _g^2 = 0.0001$, $\sigma _a^2 = 0.001$ and $\sigma _m^2 = 0.000001$;
- initial state $x_0$ (starting orientation quaternion) is computed by the QUEST algorithm (Shuster and Oh 1981) based on values of acceleration and magnetic field vector of first sample;
- sampling interval $$\varDelta t= 0.01$$;
- state covariance matrix $P_0 = I_{4x4}$.

### Results and discussion

**Fig. 3:**
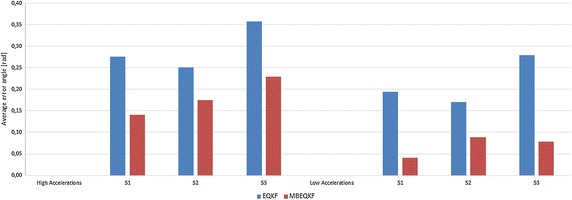
The average error angle of EQKF an MBEQKF filter showing that MBEQKF filter obtained better results for experiments with high and low accelerations

**Fig. 4:**
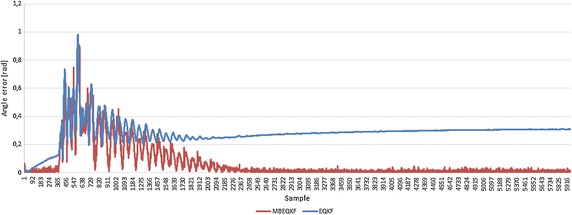
Error angle of EQKF and MBEQKF filter in segment 3 (S3) for Middle_Low capture. The MBEQKF filter better eliminates error growing in time by using kinematic dependences

**Fig. 5:**
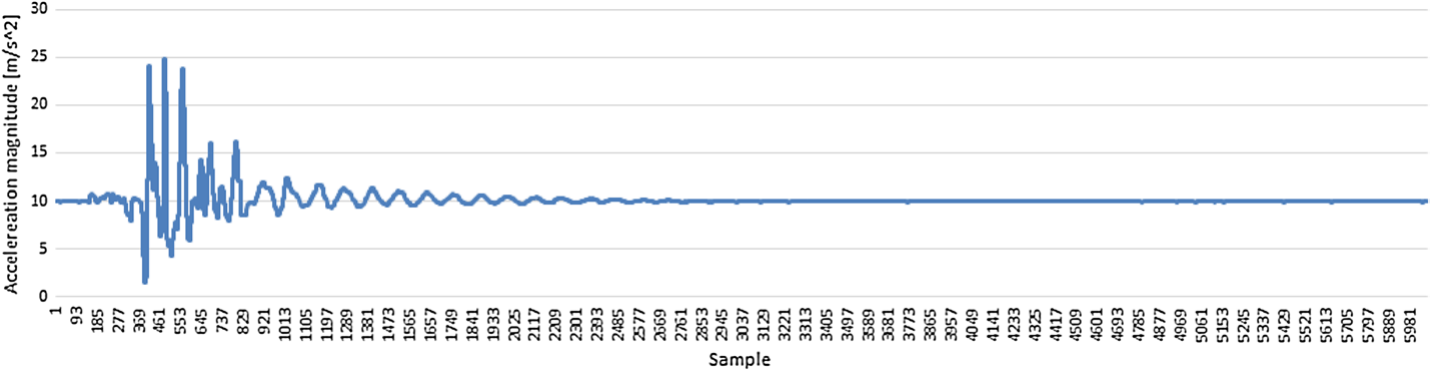
Acceleration magnitude in segment 3 (S3) for Middle_Low capture

 We performed tests for the MBEQKF with kinematic dependences and the same filter but without kinematic equations in the measurement model (EQKF) (16). In each test filter MBEQKF obtained better results than EQKF (Fig. 3). The results for high dynamic motion were always worse than that for the corresponding low dynamic tests. It is well known that a factor that strongly influences the orientation measurement is the existence and magnitude of the external acceleration of the IMU sensor. One way to manage with this is using methods of levelling the influence of linear external acceleration by, for example, an adaptation mechanism (Pruszowski et al. 2015). The highest error in each test of EQKF is always for the third segment because there are the highest accelerations. Filter MBEQKF using the kinematic chain dependences can overcome that factor. It can be seen in Fig. 4, where the maximum error is similar, but most times the error of MBEQKF is near zero value and the error does not grow in time, like it does for EQKF. The bigger error is still caused by higher acceleration values but is lower than in other filters without this mechanism (see Figs. 4, 5 for this same capture and segment).

**Fig. 6:**
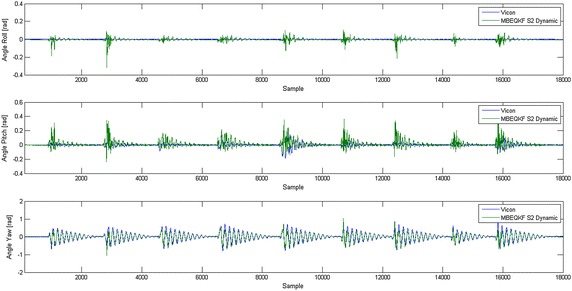
Result MBEQKF estimation of Euler angles (Roll, Pitch, Yaw) compared to angles captured by optical motion capture system (Vicon). Presented results are for segment S2 in Dynamic capture

In Table 2, the average angle errors of all tests with the pendulum are presented. The average error of the MBEQKF filter is about 6°–7° which is comparable with other solutions described in the literature. In Fig. 6 is presented the result of filter MBEQKF estimation converted to Euler angles in comparison to angles computed from optical motion capture system (Vicon).

**Table 2:** Average error angle (rad) in each segment of pendulum

Segment	EQKF	MBEQKF
S1	0.227015997	0.084566179
S2	0.202370805	0.130596741
S3	0.300123365	0.156497101

**Fig. 7:**
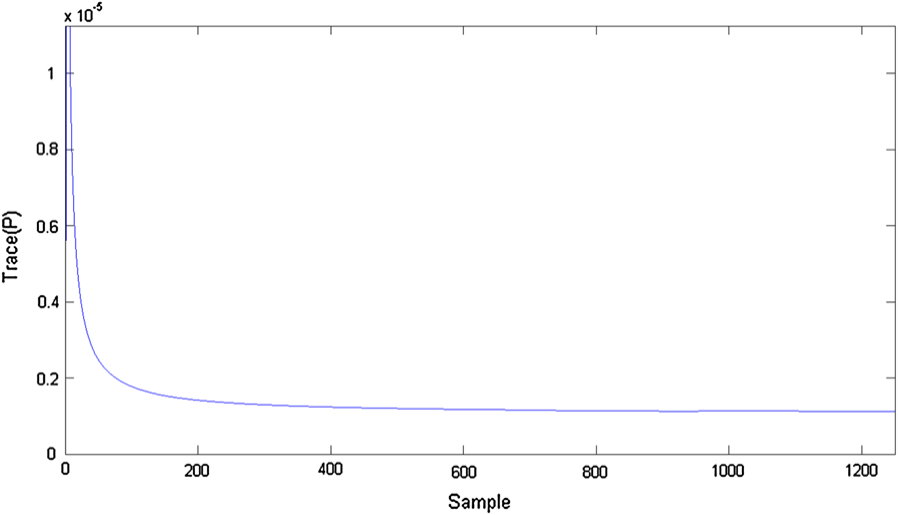
Trace of the covariance matrix (S1 for Middle_Low capture)

Examination of the trace of the error covariance matrix $P_k$, which should be minimized, can measure the convergence of the Kalman filter. This condition is fulfilled in the proposed filter (Fig. 7).

**Fig. 8:**
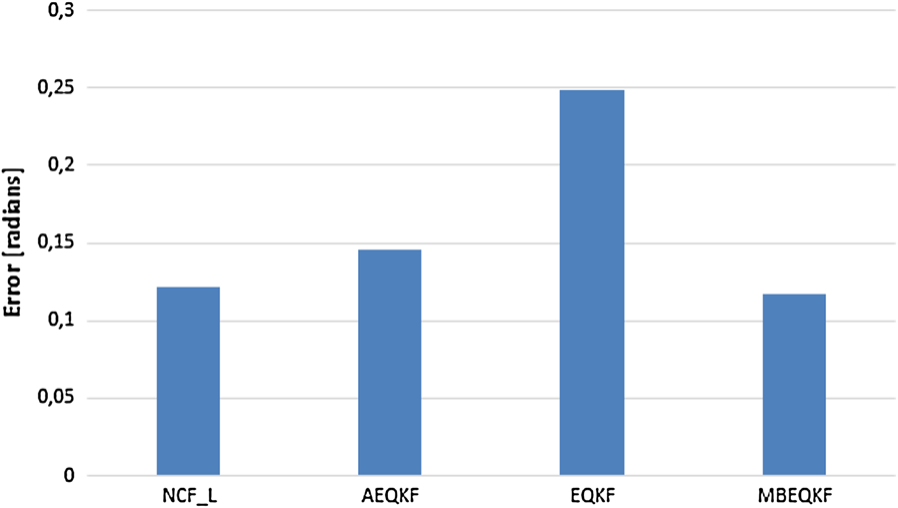
Average error angle of MBEQKF, AEQKF, EQKF and NCF_L filter

Figure 8 presents a comparison of average errors to other filters with a dynamic mechanism of levelling influence of external acceleration to orientation estimation. Filter AEQKF is an extended Kalman filter with an adaptive mechanism in which the measurement noise covariance matrix is adapted at run-time to guard against the effects of body motion. The implementation is based on Angelo (2006). Filter NCF_L is implemented based on Young (2010), where a simple complementary filter was used by passing the acceleration estimation in the skeleton model (Szczęsna et al. 2016). The proposed solution (filter MBEQKF) has the lowest error. The results are similar to the NCF_L filter because they use a similar mechanism to transfer modeled acceleration in the kinematic chain. But better results are achieved by combining this with the extended Kalman filter technique. Filter AEQKF is a Kalman filter but uses, to level the influence of high acceleration, the adaptation mechanism without using the kinematic chain dependences. In the described experiment, this led to the higher average errors.

## Conclusion

The article presents an evaluation of opportunities to improve the orientation estimation by using kinematic dependences in the kinematic chain. The results are shown for the two extended quaternion Kalman filters based on one segment (EQKF) and with kinematic dependences (MBEQKF). The results, based on experiments with the 3-segment sigle link pendulum, show a superiority of the solution based on the estimation of acceleration in the body model (skeleton), especially for child segments. The filter is universal with the small state vector and gives comparable results for an average angle error of about 6–7 degrees with other, more complex solutions presented in the literature.

## Footnotes

1
http://zgwisk.aei.polsl.pl/index.php/en/research/projects/61-repoimu.
